# Current Advance and Future Prospects of Tissue Engineering Approach to Dentin/Pulp Regenerative Therapy

**DOI:** 10.1155/2016/9204574

**Published:** 2016-03-16

**Authors:** Ting Gong, Boon Chin Heng, Edward Chin Man Lo, Chengfei Zhang

**Affiliations:** ^1^Comprehensive Dental Care, Endodontics, Faculty of Dentistry, The University of Hong Kong, Pokfulam, Hong Kong; ^2^Periodontology and Public Health, Faculty of Dentistry, The University of Hong Kong, Pokfulam, Hong Kong; ^3^HKU Shenzhen Institute of Research and Innovation, Shenzhen, China

## Abstract

Recent advances in biomaterial science and tissue engineering technology have greatly spurred the development of regenerative endodontics. This has led to a paradigm shift in endodontic treatment from simply filling the root canal systems with biologically inert materials to restoring the infected dental pulp with functional replacement tissues. Currently, cell transplantation has gained increasing attention as a scientifically valid method for dentin-pulp complex regeneration. This multidisciplinary approach which involves the interplay of three key elements of tissue engineering—stem cells, scaffolds, and signaling molecules—has produced an impressive number of favorable outcomes in preclinical animal studies. Nevertheless, many practical hurdles need to be overcome prior to its application in clinical settings. Apart from the potential health risks of immunological rejection and pathogenic transmission, the lack of a well-established banking system for the isolation and storage of dental-derived stem cells is the most pressing issue that awaits resolution and the properties of supportive scaffold materials vary across different studies and remain inconsistent. This review critically examines the classic triad of tissue engineering utilized in current regenerative endodontics and summarizes the possible techniques developed for dentin/pulp regeneration.

## 1. Introduction 

Dental pulp is often damaged by infection of cariogenic and periodontal bacteria, dental trauma, and clinical operative procedures. A routine clinical treatment for infected or necrotic pulp tissue is root canal therapy (RCT) which involves the extirpation of injured pulp and the filling of root canal systems with bioinert synthetic materials. In spite of its satisfactory clinical efficacy in infection control and pain elimination, classical treatment modalities like RCT only seek to seal the space of root canal system without restoring its original function. Therefore, various complications such as tooth fractures and reinfection occur more often in teeth that have undergone RCT due to their loss of sensory function and consequent lack of natural defense mechanisms [1]. The history of regenerative endodontics can be traced back to 1952 when Hermann unprecedentedly utilized calcium hydroxide for vital pulp therapy [2]. Apexification, a vital pulp therapy specifically indicated to treat necrotic immature permanent teeth, has demonstrated its efficacy in promoting dentinogenesis and apical closure. Nevertheless, apexification still cannot achieve continual root development or root reinforcement and is disadvantaged by its high costs and requirement for time-consuming multiple visits [3]. Such clinical challenges make it necessary to look at innovative alternative treatment modalities, with the aim of achieving partial* in situ* regeneration of dental pulp tissue or even* de novo* synthesis of revitalized whole pulp substitutes [4].

Tissue engineering, an emerging popular domain of bioscience, is a multidisciplinary research area with the ultimate aim of restoring, maintaining, and regenerating damaged/lost tissue with biologically engineered replacements through combining the principles of biology, medicine, and engineering [5]. The advent of tissue engineering has provided a brand new perspective to revitalize damaged dentin/pulp tissue using biological approaches other than traditional remedies. So far, two major strategies have been applied for dentin-pulp complex regeneration: (1) direct implantation of freshly isolated engineered cells along with/without biodegradable scaffolds and (2) implantation of preassembled tissue constructs composed of* in vitro* cultured cells and scaffolds into target anatomic location [6]. This review presents an overview of the past achievements, current advances, and future prospects in the realm of regenerative endodontics under the purview of tissue engineering. The various challenges of translating biological concepts into clinical practice will be critically examined and the focus will be on potential strategies to overcome currently intractable hurdles through the development and introduction of clinically feasible protocols.

## 2. Stem Cells

### 2.1. Dental Stem Cells

Stem cells are commonly defined as clonogenic cells that exhibit remarkable long-term self-renewal and multilineage differentiation capacity. Based on their distinction in plasticity (the ability of stem cells to give rise to different specialized cell types), stem cells can be further subdivided into embryonic stem cells (ESCs) and postnatal stem cells [7]. Although ESCs have aroused much interest, their practical application in tissue engineering and cell-based therapy is limited due to the high risk of tumorigenicity and ethical and legal issues associated with their embryonic origin. Currently, increasing research attention has been diverted to developing tissue regeneration therapy using postnatal stem cells that have been identified from various tissues and organs in human body. The term “mesenchymal stem cells (MSCs)” has been widely used to broadly describe heterogeneous fibroblastic populations of cells that possess some multilineage differentiation capacity. Nevertheless, there are concerns that it might be scientifically inaccurate to categorize all plastic-adherent cells with fibroblast-like morphology isolated from bone marrow or other tissue sources as such, since additional compelling evidence is required to support the “stemness” of unfractionated plastic-adherent cell populations. “The International Society for Cellular Therapy” (ISCR) has proposed that plastic-adherent cells, regardless of their tissue origin, be designated as “multipotent mesenchymal stromal cells” while the terminology “mesenchymal stem cells” refers to a subpopulation that fulfill specific stem cell criteria. For both cell populations, the well-recognized acronym MSCs might be applicable [8]. Dental tissues have also been investigated as potential niches of MSCs. In accordance with the minimal criteria defined by the Mesenchymal and Tissue Stem Cell committee of the ISCR, dental stem cells can be categorized as MSCs based on their properties of plastic adherence, positive or negative expression of specific surface antigen markers,* in vitro* colony formation, and trilineage mesenchymal differentiation potential [8]. Numerous studies have demonstrated that dental pulp tissue constitutes an assessable source of postnatal stem cells that can be harvested from teeth indicated for extraction or from the “disposable” exfoliated deciduous teeth with minimal invasiveness on patient donors. Up until now, various populations of postnatal stem cells have been isolated from healthy pulp tissue or its precursor apical papilla including dental pulp stem cells (DPSCs), stem cells from human exfoliated deciduous teeth (SHED), and stem cells from apical papilla (SCAP), all of which have shown great potentials for dental tissue engineering particularly in the field of dentin/pulp tissue regeneration.

#### 2.1.1. Dental Pulp Stem Cells (DPSCs)

Gronthos et al. were the pioneers that identified and characterized a stem cell population within adult human dental pulp tissue [9, 10]. Isolated from human third molars, DPSCs have demonstrated high proliferation potential, extensive self-renewal, and multilineage differentiation capacity under defined induction conditions* in vitro *[9–13]. When transplanting DPSCs with hydroxyapatite/tricalcium phosphate (HA/TCP) particles into immunocompromised mice, this cell-scaffold construct produced a dentin-resembled structure lined with odontoblastic-like cells of donor-origin accompanied with pulp-like interstitial tissue containing vascular structures [9, 14]. In recent decade, continuous attempted improvements in the transplantation methods of DPSCs into ectopic animal models have yielded numerous favorable results supporting the potential of dental pulp-derived stem cells in regenerating vascularized dentin/pulp tissue, whether or not incorporating scaffolds, whether casting scaffolds within pulp chamber of tooth slices or emptied root canals [9, 14, 15].

#### 2.1.2. Stem Cells from Apical Papilla (SCAP)

SCAP were initially isolated and characterized by Sonoyama et al. from the apical papilla, an embryonic-like soft tissue located at the apex of incompletely developed human permanent teeth [16]. Similar to the multipotency exhibited by DPSCs, SCAP have demonstrated* in vitro* osteogenic/odontogenic, adipogenic, and neurogenic differentiation capacity when induced by respective stimuli.* In vivo* studies employing animal models also demonstrated the odontogenic capacity of SCAP [16, 17]. Typical dentin structures with pulp-like tissue were observed after transplanting SCAP with HA/TCP particles into immunodeficient mice, with the dentinogenic cells at the transplantation site being verified to be of human origin. When compared with DPSCs, SCAP exhibit enhanced proliferation rate and mineralization potential. A growing body of evidence suggests that SCAP are probably a natural source of primary odontoblasts that induce root dentin formation while DPSCs appear to be the source of replacement odontoblasts [18]. Considering that apical papilla is a developing tissue recognized as a hidden niche of early progenitor/stem cells, SCAP might be superior to DPSCs for dental tissue regeneration [17, 18].

#### 2.1.3. Stem Cells from Human Exfoliated Deciduous Teeth (SHED)

SHED were first isolated by Miura et al. from vital pulp remnants of exfoliated deciduous teeth [19]. Although analogous to DPSCs which represent a specific cell population with proven capacity for self-renewal and multilineage differentiation, SHED differ from DPSCs in the expression of various surface epitopes and have demonstrated higher levels of population doublings with faster proliferation than DPSCs. These cells also exhibit greater* in vitro* differentiation potential towards the osteogenic and adipogenic lineages compared to DPSCs [20].* In vivo* transplantation of SHED loaded on scaffolds/tooth slices into immunocompromised mice has resulted in the formation of tissue that closely resembles physiological dental pulp in cellular morphology and histological architecture [21, 22]. An interesting finding by a recent study demonstrated the capacity of SHED to differentiate into angiogenic endothelium in addition to functional odontoblasts [23].

#### 2.1.4. Sorted Subpopulation of Dental Pulp Cells

Positive or negative isolation strategy is normally adopted for the selection of a specific, more homogeneous subpopulation of MSCs with optimal phenotypes. Recently, these techniques have also been reported in the fractionation of dental pulp stem cells, designed to isolate subpopulations that possess higher angiogenic and neurogenic potentials, both of which contribute to pulp homeostasis [24]. In a canine pulp amputation model, autotransplantation of CD31^−^/CD146^−^ side population (SP) cells with type I and III collagen led to the formation of pulp-like tissues with capillaries and neuronal processes by day 14 [25]. In a canine pulpectomy model, autotransplantation of both CD31^−^/CD146^−^ SP cells and CD 105^+^ cells with stromal cell-derived factor-1 (SDF-1) successfully induced complete regeneration of pulp-like tissue with vasculogenesis and neurogenesis within 14 days. This was followed by further formation of a continuous dentin-like structure along the dentinal wall of root canals [26, 27]. In this study, while the autotransplantation of unfractionated pulp cells also resulted in pulp-like tissue formation, these had lesser volume and underwent faster mineralization after transplantation when compared to fractionated dental pulp stem cells. Thus the pertinent question that arises is whether to use the sorted subpopulation or the unfractionated pulp stem cells in dentin/pulp regeneration procedure. The fact is that most of the published studies do not attempt to isolate subpopulations of dental pulp cells but directly utilize the whole heterogeneous cell population. Isolation of a purified stem cell subpopulation is futile for achieving a more homogeneous population. As soon as stem cells are isolated and plated and start to proliferate, asymmetric cell divisions will eventually lead to a mixed culture of stem cells and transiently amplifying cells that are more committed, but which are not true stem cells. Cell sorting appears not to be a cost-efficient option since there is no compelling evidence to suggest a vast improvement in facilitating dentin/pulp-like tissue regeneration.

### 2.2. Stem Cells of Nondental Origin

#### 2.2.1. Stem Cells from Bone Marrow and Adipose Tissue

Given that the availability and quality of dental pulp tissue sharply decline with age, nonodontogenic stem cells have been investigated as alternative sources among which stem cells harvested from bone marrow and adipose tissue showed greatest promise owing to their advantageous biological properties and partially shared gene expression profile of various growth factors, extracellular matrix (ECM) proteins, and transcriptional regulators [28, 29]. Ishizaka and his group isolated SP cells from pulp and adipose and bone marrow tissue of the same individual dog, respectively, and evaluated their complete pulp regeneration capacity using a pulpectomized canine tooth model [30]. Noticeably, autologous transplantation of adipose and bone marrow CD31^−^ SP cells with SDF-1 yielded tissues that were morphologically identical to that derived by transplanted pulp CD31^−^ SP cells and all three regenerated tissues possess functional properties similar to normal pulp [30]. Consistent with this finding, the mobilized stem cell subpopulation isolated from canine bone marrow and adipose tissue utilizing the granulocyte-colony stimulating factor (G-CSF) induced chemotaxis method demonstrated the complete regeneration of pulp-like connective tissues similar to pulp-derived cell subpopulations, although less in amount, angiogenesis, and reinnervation [31]. However, due to the inclusion of SDF-1 which is implicated in hematopoietic stem cell homing to the bone marrow niche via a CXC chemokine receptor-4 (CXCR4) dependent mechanism in the autotransplantation procedure, the formation of pulp-like tissue in these studies appears independent of the origin of implanted cells but was more dependent on the microenvironment at the transplantation sites [30]. Furthermore, connective tissue formation by nondental cells does not mean that it is functional. Additional evidence is needed to support the feasibility of using adipose and bone marrow tissue-derived stem cells in dentin/pulp regenerative therapies.

#### 2.2.2. Induced Pluripotent Stem Cells (iPSCs)

The generating of induced pluripotent stem cells (iPSCs) is a groundbreaking work that revolutionizes the present scenario of regenerative medicine. This can be realized through reprograming adult somatic cells or terminally differentiated cells back to a pluripotent state via overexpression of four defined transcription factors [32–34]. Analogous but superior to embryonic stem cells, patient-specific iPSCs can give rise to all cell lineages in the body, circumventing the clinical barriers of immunological rejection or ethical controversy [33, 35]. Compared to other developmentally mature somatic cells that require additional reprogramming factors, oral-derived MSCs, DPSCs, SCAP, and SHED can be more easily reprogrammed into iPSCs at higher efficiencies and are therefore a more attractive alternative source for iPSCs generation [36]. Recently, an efficient induction protocol has been developed to facilitate the differentiation of murine iPSCs (miPSCs) into neural crest-like cells (NCLC)* in vitro* [37]. These NCLC further demonstrated their potential to differentiate into dental mesenchymal cells including odontoblasts upon coculture with mouse dental epithelium. Furthermore, an* in vivo* study demonstrated the formation of dentin-like and dental pulp-like structures upon transplantation of the reconstructed tooth germs derived from miPSCs together with epithelium and mesenchyme into mouse subrenal capsule [38]. Despite that, not all of the reconstituted tooth germs produced perfect tissue-engineered tooth-like structures. These interesting findings demonstrate much potential of iPSCs in future regenerative dentistry research.

## 3. A Call for Dental Stem Cell Banking System

When cell-based therapy is adopted for dentin/pulp tissue regeneration, the procuring of a readily available stem cell source is of the highest priority. While the favorable properties of low immunogenicity and immunosuppression exhibited by oral-derived adult stem cells offer much possibility for allogeneic cell transplantation, these adult stem cells will likely express histocompatibility antigens upon differentiation and will thus not be able to sustain the immunosuppressed state [39–41]. Hence, the majority of cell-based approaches still prefer an autologous cellular source for transplantation to best mitigate the risks of immunological rejection and pathogenic transmission [42]. However, autologous cell sources are often not necessarily off-the-shelf at the time of treatment. The isolated small quantities of mesenchymal-like stem cells from pulp tissue have to undergo long and extensive processing prior to utilization in transplanting procedure, which prompts the need for a well-established “dental stem cell banking” system for cell isolation and long-term storage. In recent decades, while increasing numbers of private tooth banks have been established, none of them provide clinical-grade MSCs due to a lack of affordable good manufacturing practice (GMP) compliant facilities on the market [42, 43]. Before establishing a public dental stem cell banking system, the state and quality of preserved cells should be strictly evaluated and monitored because the origin of stem cells varies and an array of confounding factors such as donor-associated variability is likely to influence the overall therapeutic efficacy.

### 3.1. Is Inflamed Dental Pulp Tissue Eligible?

Conventionally, infected or inflamed pulp tissue will be completely removed by pulpectomy in order to create a sterile environment for the subsequent restorative treatment. However, a considerable portion of healthy and vital pulp tissue might also be discarded as medical waste together with the necrotic dental pulp. Recently various studies have reported successful isolation of viable stem cells from inflamed dental pulp, but with conflicting regenerative effects on dentin/pulp tissue. Yazid et al. showed that MSCs obtained from primary inflamed pulp exhibited highly dysfunctional MSCs stemness and minimal immunoregulatory capacity [44]. By contrast, Pereira et al. demonstrated that stem cells derived from normal pulp tissue resembled that from inflamed pulp in morphological characteristics, proliferation, and differentiation potentials [45]. Yu et al. reported that stem cells from normal and inflamed pulp exhibited similar proliferation rates and multipotency [46]. Using a severe combined immunodeficient mice model (SCIDM), Alongi et al. observed that dentin/pulp-like complexes formed by dental pulp stem cells from inflamed pulp (DPSCs-IP) resembled the tissues formed by DPSCs from normal pulp [47]. While inflammation has been identified to facilitate odontoblastic differentiation and dentin matrix formation, severe inflammation is known to induce cellular apoptosis [48]. Although DPSC-IPs appears to be a more readily available cell source, the therapeutic efficacy still awaits being verified by further investigations and the risk of pathogenic transmission from infected tissues arouses much concern [47].

### 3.2. Does Donor's Age Matter?

The right time for banking cells is a thought-provoking question. There seems to be a noticeable progressive age-associated reduction in regenerative capacity of MSCs present in multiple adult organs including teeth [49]. Molecular differences in cell population of different ages have been detected with more robust expression of genes implicated in cell stemness, mitosis, and proliferation in deciduous teeth than in permanent teeth [50]. Conversely, mobilized DPSCs (MDPSCs) with granulocyte-colony stimulating factor (G-CSF) derived from young dog demonstrated similar proliferation, migration potential, and antiapoptotic effects as well as similar expression levels of genes encoded for trophic factors to that from aged dog [51]. In that study, after autologous transplantation of these two different age groups of canine MDPSCs into pulpectomized teeth, pulp tissues produced by aged MDPSCs demonstrated significantly retarded growth compared to young MDPSCs, indicating that it might not be MDPSCs per se but the resident endogenous MSCs supported by MDPSCs-derived trophic effects that contributed to the observed age-dependent decline in pulp regenerative capacity [51]. When comparing DPSCs obtained from third molars of patients ranging from 12 to 30 years old, Kellner et al. concluded that age appeared not to be a crucial determinant of the maximal cell division potential and that donors ranging from 12 to 30 years old were all suitable groups for stem cell banking [52].

### 3.3. Considerations for Other Confounding Factors

In addition to the physical state of dental tissue, other general health-associated parameters like smoking and nutrition are possible factors that can influence the physiological activity of dental pulp cells (DPCs) and overall biological properties of dental pulp tissue, although the evidence is weak [53, 54].

## 4. Scaffold

A scaffold is one of the three essential components of classic tissue engineering strategy. Functionally, scaffolds provide a three-dimensional (3D) temporary structural framework for cell seeding, adhesion, proliferation, and spatial distribution until their complete replacement by the newly synthesized matrix of endogenous host cells [55, 56]. Currently, the role of an ideal scaffold is not limited to serving as a bioinert cell delivery vehicle but extends to acting as a bioactive platform regulating cellular activities and intracellular communication and thus to faithfully recapitulating their physiological microenvironment-ECM.

Collagen, a major macromolecular constituent of dentin ECM with excellent biocompatibility, is the most extensively studied naturally occurring material for dental tissue engineering. Comparison of different natural polymers including collagen type I, collagen type III, chitosan, and gelatin suggests that hDPCs plated on collagens, particularly type I collagen, perform best in terms of proliferation and mineralization capacity [57]. Combined utilization of a collagen scaffold carrying DPSCs and dentin matrix protein 1 (DMP1) at a simulated furcal perforation site within dentin slice resulted in apparent pulp-like tissue organization after transplantation into a SCIDM [58]. A recent study demonstrated the positive effects of collagen in supporting the survival and odontogenic differentiation of SHED* in vitro* and also facilitating pulpal tissue regeneration in a full-length root canal* in vivo* [59]. When cultured in a 3D-ECM-embedded type I collagen/chitosan scaffold, the expressions of putative odontogenic differentiation markers in DPSCs were upregulated with crystalline hydroxyapatite nucleation whereas only amorphous calcium phosphate nucleation occurred with DPSCs cultured in ECM-free scaffold. Furthermore, formation of pulp-like tissue expressing dentin sialoprotein (DSP) and dentin phosphophoryn (DPP) was detected in a study after transplanting ECM scaffolds containing DPSCs into SCIDM [60].

Synthetic materials such as polylactic acid (PLA), polyglycolic acid (PGA), and their copolymer poly lactic-glycolic acid (PLGA) have attained approval from the United States Food and Drug Administration (FDA) for wide application due to their nontoxic properties. An earlier study demonstrated that PGA accelerated collagen deposition and appeared to be more conducive to DPCs proliferation compared to alginate or collagen scaffolds [61]. A highly desirable outcome has been reported when using poly-D,L-lactide/glycolide (PLG) scaffolds for delivery of SCAP and DPSCs into target transplantation site. The results showed* de novo* regeneration of vascularized pulp-like tissue in an empty root canal space accompanied by deposition of dentin-like tissues along dentinal walls formed by newly derived odontoblast-like cells [62].

Platelet-rich plasma (PRP) and platelet-rich fibrin (PRF) are other potential materials easily obtained from autologous blood and thus can minimize the risk of adverse immunological response. These can release many vasculogenesis-associated factors including vascular endothelial growth factor (VEGF), platelet-derived growth factor (PDGF), basic fibroblast growth factor (bFGF), insulin-like growth factor-1 (IGF-1), transforming growth factor-b *β*1 (TGF-b *β*1), and TGF-b *β*2, when platelets are activated [63, 64]. Recently, PRF and PRP at appropriate concentrations have been demonstrated to induce proliferation and accelerate mineralized differentiation of DPSCs [63, 65].

In a nutshell, although both natural polymers and synthetic materials have demonstrated promising therapeutic effects on dentin/pulp regeneration, neither of them embraces all the favorable properties that can recapitulate the cellular physiological microenvironment. Natural polymers are usually afflicted with issues of purity and antigenicity. Synthetic polymers, albeit circumventing the potential risks of immunoreactivity and being more readily amenable to clinical applications with tailor-made physiochemical properties, still lack bioactive molecules present in native ECM that are necessary for cellular recognition and interaction, in addition to various technical challenges in regulating pore sizes and achieving high porosity structures [66].

Recently, nanofibrous polymer scaffolds have been synthesized to biomimic ECM in structure and function. They appear superior to other scaffold materials at microstructural level or other morphological arrangements owing to their interconnected porous networks and advantages in high surface area to volume ratio [4]. Most strikingly, nanofibrous polymers can be tailor-made to facilitate specific cellular behaviors and cell-ECM interaction [67, 68]. To date, only three cutting-edge techniques are available for fabrication of nanofibrous scaffolds, which are molecular electrospinning, self-assembly, and thermally induced phase separation (TIPS). Electrospinning process that involves the application of a high voltage to a polymer solution or melt loaded within a needle orifice/syringe can generate continuous polymer fibers with diameters varying from nanometers to micrometers [69, 70]. The latest research milestone of electrospinning technique lies in its new role as drug delivery vehicles rather than a single cell carrier only. Interestingly, novel antibiotic-containing nanofibrous scaffolds not only create a sterile environment conducive to tissue survival and development but tend to be more cell-friendly by minimizing toxic side effects via controlled release of chemical agents at lower concentrations [71, 72]. TIPS system refers to the induction of a homogenous polymer solution to separate into a polymer-rich phase and a solvent-rich phase by lowering the temperature to a critical point. TIPS has demonstrated synergistic effects with other techniques to generate 3D nanofibrous scaffolds with interconnected pore structures that can facilitate cell infiltration, cell proliferation, nutrient distribution, and tissue formation [73, 74]. The natural self-assembly process, regarded as the spontaneous organization of components into a particular structure, can be mimicked and utilized to produce nanofibrous polymers from engineered self-assembling peptides [67, 75]. Self-assembly at the molecular level contributes to hydrogel formation which has demonstrated unique cell encapsulation function and superior capacity to promote cell adhesion, proliferation, and differentiation [76–78].

## 5. Limitation of Cell Transplantation Strategy in Preclinical Animal Models

Several preclinical animal models based either on ectopic or on orthotropic transplantation approach have been developed to translate cell-based therapy into clinical reality. Although a substantial body of evidence has confirmed the regeneration of mineralized tissue, some studies still failed to detect the presence of dentin-like tissue but instead found tissues with bone-like morphology [79]. This is likely attributed to the different microenvironment of the transplantation sites in animal studies which appears to be a fourth element of tissue engineering that influences the fate of stem/progenitor cells* in vivo*. Comparisons of data extracted from different studies suggest that the fate of implanted stem/progenitor cells may be site-associated rather than being influenced by their origin, with a tendency to differentiate into the odontoblast phenotype when transplanted into the pulp chamber but into other cell lineages when transplanted into other target locations [80]. SCAP, a potential cell source for* in situ* dentin/pulp regeneration, may be lost during the transplantation process. It cannot be ruled out that the quiescent host stem cells residing in the niche of the transplantation site may be stimulated and recruited into the regenerated construct together with pulp-derived stem cells and thus pulp cell may lose its predominant effect on tissue mineralization [80]. Additionally,* in situ* pulp regeneration is often disadvantaged by ischemic conditions due to the constricted opening at the root apex allowing for blood microcirculation while root fragments or tooth slices rarely face this problem. Despite the deficiency of ectopic models in mimicking the* in situ* tooth microenvironment in clinical practice, the implementation of orthotropic model encounters formidable challenges. After transplantation into the pulpectomized teeth, the fabricated tissue replacement has a tendency to adhere to the coronal portion of root canals, leaving the middle and apical parts incompletely filled [78]. This is mainly due to the unique anatomical structure of root canals, with progressively complex spatial patterns and increased spatial pressure towards the apex, which in turn constricts the placement and extension of any replacement. In an animal study, Iohara et al. successfully initiated a complete orthotopic pulp regeneration procedure in a dog model using autologous pulp CD105+ cells. Two weeks after implantation of CD105+ cells with stromal cell-derived factor-1 (SDF-1) into pulpectomized canine mature teeth, pulp-like tissue with vasculogenesis and neogenesis filled the empty root canal space accompanied by the deposition of dentin-like structure along the dentinal walls [26]. In addition, studies applying a mixture of DPSCs and PRP to canine teeth after pulpectomy demonstrated partial vital pulp regeneration even though the typical structures in most cases have been identified as periodontal-like tissue [81, 82].

## 6. Potential Strategies to Promote Dentin/Pulp Regeneration 

The critical requirements for successful dentin/pulp regeneration are not confined to the morphogenesis of dentin/pulp-like tissue but should also be accompanied with angiogenesis and neurogenesis. It must be noted that the deposited dentin-like tissues on existing dentin structure are produced by the newly differentiated odontoblasts from the dentinal wall within the root canal space. Currently, potential strategies dedicated to optimizing stem cell-mediated dentin/pulp regeneration are directed towards two main objectives: firstly angiogenesis induction and, secondly, the promotion of tissue mineralization.

The long-term survival of tissue-engineered implants largely depends on rapid establishment of adequate blood supply at the graft sites which functions as a conduit for nutrient transportation, oxygen delivery, and metabolic waste excretion. This principle is particularly relevant to dentin/pulp regeneration given that the opening at root apex is too constricted to allow for intracanal blood infusion [56]. Due to a delay in nutrition and oxygen support, the therapeutic efficacy of stem cells during long-term engraftment may be reduced dramatically and implanted cells may ultimately enter into hypoxia-induced apoptosis. Conventionally, it is routine to await the access and ingrowth of new vasculature into the transplanted replacement within the canal space. This approach, however, may be problematic when fabricating a cell-dense thick construct which blocks the diffusion of essential oxygen and nutrients. In addition, it is highly probable that prior to their integration with host tissue the bioengineered replacement grafts will suffer from ischemia and thus undergo necrosis since the complete establishment of host-derived vascular networks including initial ECs migration, proliferation, angiogenic sprouting, and final phase of vascular stabilization is quite a lengthy process [83]. Prevascularization has emerged as a viable alternative strategy to address this challenge. With the assistance of the vessel plexuses formed in advance, the bioengineered grafts may more rapidly anastomose with the host-derived vascular structure after transplantation. Several prevascularization approaches in attempting to facilitate dentin/pulp regeneration will subsequently be discussed in detail.

### 6.1. Hypoxia for Angiogenesis

Hypoxia is a common phenomenon encountered by dental pulp tissue under both pathological and nonpathological conditions. Due to their unique anatomical structure that is surrounded by hard dentin with only a narrow opening at root apices, DPCs are typically susceptible to ischemia during traumatic injuries when surrounding vascular bundles are severely ruptured or during restorative treatment whereby local anesthetics containing vasoconstrictors slow down the velocity of blood microcirculation. Exposure to ischemia blocks the oxygen influx transported by the blood stream and thus disrupts the oxygen homeostasis of dental pulp [84]. In addition, the progressive inflammatory reactions resulting from dental caries usually increase intracanal pressure and thus force oxygen out. Even under nonpathological conditions, the oxygen tension within the root canal space (approximately equivalent to 3%) appears lower than that in ambient air where* ex vivo* pulp cell culture and expansion are normally conducted. For these reasons, some investigators have tried to simulate the conditions of pulp hypoxia and explore how DPCs respond to low oxygen levels. Amemiya et al. demonstrated that canine DPCs under hypooxygenation conditions exhibited higher formazan production and growth rate compared to pulp cells incubated under normoxic conditions [84]. Subsequently, an array of studies conducted on hDPCs reported that hDPCs cultured under hypoxic conditions exhibited increased proliferation rate, higher percentages of side populations, and enhanced angiogenic potential as well as increased erythropoietin expression* in vitro* than those incubated under normoxic conditions [85, 86]. Lida et al. successfully applied hypoxic conditions for isolation of hDPCs from inflamed teeth of older patients and demonstrated their odontogenic capacity both* in vitro* and* in vivo* [87]. Consistent with these findings, a recent study demonstrated extensive vessel-like networks being formed in SCAP-HUVEC coculture group under hypoxic conditions and that the induced hypoxic environment significantly upregulated the expression of HIF-1*α*, ephrinB2, and VEGF [83]. Collectively, hypoxia may be a viable and efficacious approach to enhance the probability of isolating living DPCs from damaged or aged pulp tissues and to prime the angiogenic capacity of DPSCs for pulp repair and regeneration.

### 6.2. Combinatorial Cell Seeding

In recent years, many research groups have succeeded in vascularizing artificial tissue constructs via codelivery of ECs with MSCs of various types. The significance of coculture system is based on the speculation that intricate signaling cross talk may be established among heterogenous cell populations, possibly regulated by direct cellular interaction or paracrine signaling [88]. This hypothesis is also applicable to tooth germ development during which reciprocal cross talk between epithelium and mesenchyme orchestrates sequential cellular activities including cell proliferation, differentiation, and even overall homeostasis, all of which play a crucial role in tooth morphogenesis [89, 90]. The original intention of using coculture systems in pulp regenerative therapy appears mainly to potentiate committed differentiation of DPSCs towards the osteo/odontoblast lineage. So far, a wide variety of cell candidates have been attempted in cocultivation with DPSCs. The coculture of DPSCs with dental ECs enhanced the odontoblastic differentiation of DPSCs, possibly via paracrine signaling of BMP2 and BMP4 secreted by dental ECs [91]. When cocultured with osteoblasts, expression of osteogenic-related genes has been shown to be upregulated with increased mineralization in DPSCs [92]. Recently, the selection of cell types incorporated into coculture systems has been driven by angiogenesis/vasculogenesis consideration to a greater degree. The signaling events at the molecular and cellular levels are tightly orchestrated by the interplay between pericytes and ECs. Without the guidance of pericytes, the further maturation of blood vessels will be impeded and the established vascular structures will not be maintained. Previous studies demonstrated that DPSCs resided in the microvascular niche of dental pulp tissue, which was supported by the expression of *α*-smooth muscle actin and several mesenchymal and endothelial cell markers by perivascular cells [93]. Dissanayaka et al. initiated the direct coculture of DPSCs and ECs* in vitro* and found that HUVECs dramatically facilitated the osteogenic/odontogenic differentiation of cocultured DPSCs compared with either DPSC-alone or HUVEC-alone cultures. In turn, DPSCs stabilized and increased the longevity of the preexisting HUVEC-formed vasculature [94]. DPSCs have been identified to be located adjacent to blood vessels like a sheath covering HUVECs, analogous to the spatial relationship and interaction between pericytes and ECs [78, 94]. Consistent with this finding, Janebodin et al. demonstrated the superiority of coculture of ECs with murine DPSCs in forming more mature* ex vivo* tubular-like networks than coculture of ECs with bone marrow mesenchymal stem cells (BMMSCs). Interestingly, this study not only proved the pericyte-like topography and potential function of DPSCs but also identified that the mechanism of DPSCs in angiogenic induction was VEGFR-2 dependent [95]. Another* in vitro* study demonstrated that coculture of SCAP and HUVEC under hypoxia enhanced the formation of vessel-like structure with increased tubular length and branching points and that the secretion of VEGF by SCAP might be utilized by HUVECs [83]. Further* in vivo* study implied that contribution of DPSCs to vascular morphogenesis when coimplanted with HUVECs not only was confined to the paracrine effects of secreted proangiogenic factors like VEGF, but also involved cell migratory mechanisms and corresponding scaffold disruption [78]. While promising, the combinatorial culture of pulp-derived stem cells with HUVECs seems not applicable to clinical settings due to the immune incompatibility involved in HUVECs utilization. Therefore, potential alternatives like endothelial progenitor cells (EPCs) and human dermal microvascular endothelial cells (HDMEC) should be further investigated in combination with pulp stem cells.

### 6.3. Scaffoldless Delivery Approach with Cell Sheet Technology (CST)

Conventionally, stem cells are combined with exogenous scaffold materials to form a 3D engineered structure before transplanting into target sites. However, in addition to those biomaterial-associated problems, this well-recognized technique has long been questioned due to the inadequate cell migration and cell retention within the supporting scaffolds. Above all, the aforementioned biomaterials are far from being able to replicate the natural ECM or the surface of implantable device. A decade ago, Iohara et al. pioneered a 3D* in vitro* culture system specifically for DPCs [96]. Unlike commonly used protocols, this system intended to harvest cell pellets or aggregates simply via one-step centrifugation. Compared to the monolayer culture, significantly higher ECM accumulation and odontogenic differentiation were observed in the 3D pellet. After being treated with rh-BMP2, the transplantation of DPCs pellet successfully induced reparative dentin formation in amputated canine tooth pulp [96]. Considering that the centrifugation step may bring extra force to affect cellular behaviors, Syed-Picard et al. engineered more advantageous 3D scaffoldless tissue instead, by using a self-assembly system where cells could arrange and control their preferred 3D microenvironment all by themselves. Following insertion into the canal space of human tooth root segments, this 3D self-assembled construct regenerated vascularized dental pulp-like tissue in SCIDM [97].

Recently, a similar but different innovative “scaffold-free” approach developed for regenerative medicine, designated as “cell sheet technology” (CST), has become a popular topic of active research in the field of periodontal tissue engineering [98–100]. Superior to routine cell isolation methods utilizing dissociative enzymes or mechanical forces, both of which probably compromise cell viability, CST is a noninvasive approach relying on a thermoresponsive polymeric material, referred to as poly(N-isopropylacrylamide) (PIPAAm). Cells plated on PIPAAm-treated surface can be detached as a contiguous monolayer with intact structure and ECM components simply by using a modest temperature drop [101, 102]. However, owing to the poor manipulability of cell sheets in a narrow tubular space, the graft is prone to undergo necrosis without adequate ECM deposition and nutrition acquisition. Na et al. modified the cell sheet structure to a more flexible 3D pellet system-cell sheet derived pellet (CSDP) and achieved pulp-assembled tissue regeneration in an entire empty tooth canal space after ectopic transplantation into SCIDM [103]. Despite promising results, additional comprehensive* in situ* evaluation of scaffoldless delivery system is required in large animal models.

### 6.4. Injectable Scaffold Delivery System

The introduction of preengineered 3D tissue constructs into transplantation sites requires a viable and effective delivery system. In general, scaffold adopted for bone or another body tissue regeneration is often fabricated with a rigid structure in order to provide sufficient physical support. Proof-of-principle transplantation experiments have provided valuable mechanistic information for dentin/pulp regeneration when using relatively rigid scaffolds like PLLA casts with tooth slices or fragments to deliver pulp stem cells [104]. However, theoretically, extra support is not necessarily required from engineered replacement since the surrounding dentin alone can offer adequate physical and structural support and the unique complex anatomical structure of root canals—a narrow and tapering shape from the coronal to apex—hampers the insertion of stiff biomaterials. Furthermore, the implanted pulp cells within the rigid scaffolds will encounter great difficulty in attaching and penetrating into the dentinal walls, which impedes the generation of functional odontoblasts and production of new dentin. Encouragingly, self-assembly techniques at the molecular level contribute to the formation of an injectable polymer hydrogel that closely mimics the ECM structure at the nanoscale level [105]. Similar to a living tissue, the hydrogel possesses a higher water content and lower interfacial tension with good consistency. Extra superiority originates from its soft property, with minimal invasiveness to the surrounding dentin or the canal structure. This hydrogel created from multidomain peptides allows for engineered constructs administered in a soft 3D matrix and delivery into the root canal system by a syringe with a prolonged duration of action. Galler et al. demonstrated that DPSCs encapsulated in a self-assembled peptide hydrogel exhibited higher survival rates, enhanced spreading, and migration ability than nonbioactive gels. Upon incorporation of inductive GFs within the scaffold, it was demonstrated that only DPSCs encapsulated hydrogel replete with GFs expressed dentin-specific markers and formed well-vascularized soft connective tissue analogous to normal functional dental pulp after ectopic transplantation [77]. Similar findings were also reported when SHED were mixed with Puramatrix—a commercially available peptide hydrogel—and injected into full-length root canals or transplanted into immune-deficient mice. Moreover, the microenvironment created by this type of hydrogel at 0.15% concentration can stimulate not only the survival, proliferation, and functionalization of DPSCs but also the vascular morphogenesis of HUVECs [78]. This novel type of delivery system, while highly promising, has obvious flaws in lack of control over tissue formation and development. Currently, efforts are devoted to making hydrogel photopolymerizable and researchers are still looking for novel self-polymerizable biomaterials which are in the liquid state but can be transformed into a rigid form once delivered into the target sites [106].

### 6.5. Inductive Growth Factors

The lineage commitment and differentiation of dental stem cells during dentin/pulp tissue repair and regeneration markedly differ depending on multiple factors. Apart from their intrinsic gene expression and epigenetic profiles, the cellular fate of dental pulp-derived stem cells seems more likely to be determined by the bioactive signaling molecules within the local microenvironment [107, 108]. Currently, a diverse array of inductive growth factors have been identified implicated in important cellular events during dentinogenesis and pulp tissue regeneration and they are either (1) released from the degraded dentin matrix [109] or (2) derived from the etched platelets within induced blood clot during revitalization procedures or (3) artificially synthesized* in vitro* and locally delivered into engineered tissue.

Nakashima demonstrated recombinant human BMP2 and BMP4 (rh-BMP2, rh-BMP4) induced tubular dentin and osteodentin formation on canine amputated tooth pulp when capped with inactivated dentin matrix [110, 111]. Studies conducted by Casagrande et al. unequivocally demonstrated that SHED seeded on EDTA-treated tooth slice/scaffolds displayed stronger expression of putative odontoblastic differentiation markers than the untreated group and indicated dentin-derived BMP2 is required to induce odontolineage differentiation of SHED [112]. VEGF is a prototypic proangiogenic factor and a potent ECs mitogen with profound regulatory effects on signaling cascades implicated in angiogenesis/vasculogenesis [113, 114]. The truncated human dental pulps showed increased microvessel density and neovascularization after treatment with rh-VEGF for 7 days [115]. VEGF has been demonstrated to induce SHED into angiogenic endothelium both* in vitro* and* in vivo* [23]. In an* in vitro* study in which SCAP were cocultured with HUVECs, VEGF secreted by SCAP were metabolized by HUVECs to facilitate the formation of new vasculatures and the maturation of sprouted capillary networks [83]. bFGF has been demonstrated to regulate odontogenesis and is released by dental pulp fibroblasts upon injury [116, 117]. It has been revealed that bFGF potentiates proliferation and migration of human DPCs (hDPCs) while it suppresses their ALPase activity and mineralization [118]. Notably, the inhibiting effect of bFGF on cytodifferentiation of hDPCs was relieved when bFGF-pretreated hDPCs were in subsequent culture without bFGF [118]. Recently, an* in vivo* study demonstrated that bFGF increased the survivability of DPSCs seeded on silk fibroin scaffold and resulted in the formation of pulp-like tissue replete with vasculature formation and calcium mineral deposition along the radicular dentinal wall after transplantation into SCIDM [119]. However, despite these promising outcomes, the selection of growth factors, the variation in their dosage, and the time to deliver them into target sites may exert entirely different biological effects on cellular behaviors, ranging from induction to inhibition and even cell apoptosis.

### 6.6. Gene Therapy

Loading scaffolds with bioactive drugs or protein molecules intended for promoting dental tissue regeneration has achieved favorable results [120]. Nevertheless, it is highly probable that the harsh conditions for manufacturing scaffolds including extremes of pH, heat, shear strength, and the introduction of toxic solvents might compromise the bioactivity of any signaling molecules incorporated within the scaffolds. While a novel technique named “ultrapurified water freeze-drying” can overcome these shortcomings and successfully regenerate tissue that morphologically resembled normal dental pulp, the local delivery of growth factors into root canals still remained a challenging task considering the large dosages required and repeated administration due to their short active half-life coupled with poor distribution [121]. Gene therapy, a relatively new frontier of medicine, has emerged as a viable and promising strategy that obviates the delivery of exogenous molecules. With this approach, genes encoding the desired signaling molecules including growth factors, morphogens, transcription factors, and ECM molecules are delivered by viral or nonviral vectors into target somatic cells to induce a series of biological responses that contribute to the regeneration of the target tissue. Yang and colleagues successfully transfected rat STRO-1 selected DPSCs with the human BMP-2 gene by utilizing adenoviral vectors. They demonstrated that BMP-2-transfected DPSCs secreted BMP-2 at a high level and underwent more effective induction towards the odontoblast phenotype compared to nontransfected cells and produced calcified ECM without external addition of osteoinductive factors into the culture medium [121]. Their subsequent* in vivo* study further confirmed the efficacy of BMP-2 transfection therapy in potentiating tissue mineralization within the regenerated construct in favor of bone-like structure rather than dentin-like morphology [79]. However, a literature search found a lack of evidence in this field and gene therapy has yet to prove its efficacy in rescuing necrotic tooth pulp as part of dentin/pulp regenerative treatment [56]. Additionally, potential health hazards still exist due to the infective ability of viral vector systems although they can be modified to reduce their detrimental effects to the host cell. There remains much to be accomplished including the safety issues and the accurate control of gene delivery prior to the approval by FDA of gene therapy for clinical research in endodontic treatments.

## 7. Conclusion 

Owing to the recent advances in the field of biomedicine, tissue engineering, and material science, great progress has been achieved in the development of regenerative endodontics. We are now at a stage of paradigm shift in endodontic treatment from traditional restoration to complete replacement of the compromised dental pulp tissues which can be realized by two scientifically meritorious approaches, cell transplantation and cell mobilization, including revitalization/revascularization procedure. While simple to operate, the cell mobilization approach may not be suitable to tissues with large size defects, especially in the circumstance of* de novo* pulp regeneration in the full-length root. In addition, cells trapped within fibrin clots during revitalization/revascularization have neither a definite origin nor a predictable quantity and thus the phenotype of regenerated replacements filling in root canals cannot be well characterized, leading to variations in overall therapeutic efficacy. In comparison, cell transplantation based on the utilization of the meticulous triad of classic tissue engineering including stem cells, scaffolds, and signaling molecules has achieved promising outcomes. This cell-based method is capable of repairing tissue defects with an extensive size [43, 122]. Despite these remarkable accomplishments, cell transplantation strategy still faces significant challenges and has not demonstrated being a clinically feasible mode of treatment. The stem cell banking procedure involving cell isolation,* ex vivo* cell manipulation, and ultimate shipping back to practitioners is a rather time-consuming process and the cost involved may be too excessive over relatively mature alternative therapies like dental implants or RCT. During the expansion period, cell senescence, loss of stemness, and microbial contamination are likely to occur, leading to biological deterioration and even cellular apoptosis. Worse still, MSCs are prone to experience malignant genetic transformation and thus arouse safety concern of tumorigenesis [42]. If these barriers can be resolved, stem cell-mediated transplantation would be a very much sought-after treatment modality for dentin/pulp tissue regeneration.

To date, no randomized clinical controlled trials have been conducted to compare the efficacy of cell mobilization or cell transplantation therapy and traditional endodontic treatment. Superior to tooth restoration, regenerative therapy is considered as a potential solution to address the deficiencies in tooth homeostasis, immune defense system, blood supply, and functional dentin-pulp complex formation. Moreover, it prolongs the life of natural dentition, defers the extraction fate of teeth with partially necrotic pulp, and thus ultimately improves the overall oral health-related quality of life. Collectively, additional studies in these fields should continue in order to work out a clinically translatable platform on dentin/pulp regeneration. Future research prospects in regenerative endodontics rely on the multidisciplinary collaboration of clinicians, biomaterial scientists, and engineers with expertise in their own fields and their joint contributions to the development of dentin/pulp regeneration therapy.
